# Influence of the experimental design of gene expression studies on the inference of gene regulatory networks: environmental factors

**DOI:** 10.7717/peerj.10

**Published:** 2013-02-12

**Authors:** Frank Emmert-Streib

**Affiliations:** Computational Biology and Machine Learning Laboratory, Center for Cancer Research and Cell Biology, School of Medicine, Dentistry and Biomedical Sciences, Faculty of Medicine, Health and Life Sciences, Queen’s University Belfast, Belfast, UK

**Keywords:** Gene regulatory networks, Statistical network inference, Gene expression data, Experimental design, Interventional data

## Abstract

The inference of gene regulatory networks gained within recent years a considerable interest in the biology and biomedical community. The purpose of this paper is to investigate the influence that environmental conditions can exhibit on the inference performance of network inference algorithms. Specifically, we study five network inference methods, Aracne, BC3NET, CLR, C3NET and MRNET, and compare the results for three different conditions: (I) observational gene expression data: normal environmental condition, (II) interventional gene expression data: growth in rich media, (III) interventional gene expression data: normal environmental condition interrupted by a positive spike-in stimulation. Overall, we find that different statistical inference methods lead to comparable, but condition-specific results. Further, our results suggest that non-steady-state data enhance the inferability of regulatory networks.

## Introduction

More than ten years after the completion of the Human Genome Project (Consortium, 2004; Lander et al., 2001; Venter et al., 2001) it is nowadays generally acknowledged that in order to obtain a functional understanding of organisms and the emergence of their phenotypes it is not sufficient to study sequence data alone. Instead, within recent years there are increasing attempts to infer genome-scale molecular interactions from high-throughput data to tackle this problem. Depending on the applied technology, this resulted in the construction of protein–protein interaction networks, metabolic networks or transcription regulatory networks (Blais & Dynlacht, 2005; Förster et al., 2003; Lee et al., 2002; Ma et al., 2004; Palsson, 2006; Yu et al., 2008). These networks can be considered as *phenomenological networks* because each interaction within these networks is based on the measurement of the corresponding biochemical binding between genes or gene products. For examples, in a transcriptional regulatory network an edge in the network corresponds to the binding of a transcription factor to the promotor region of the DNA that is necessary to regulate the transcription of a gene. Or in protein–protein interaction networks an edge corresponds, e.g., to the binding of two proteins to form a protein complex. In contrast to these *phenomenological networks* gene regulatory networks constructed from gene expression data are *inferential networks*. The difference is due to the nature of the employed data to construct the network because gene expression data do only provide information about the concentration of mRNAs, but not direct information about the biochemical binding of genes or gene products. For this reason, an edge in a gene regulatory network is not uniquely specified but could correspond either to transcription regulation, as in transcriptional regulatory networks, or to protein bindings, as in protein–protein interaction networks (de Matos Simoes, Tripathi & Emmert-Streib, 2012). In the remainder of this paper we focus on gene expression data and the gene regulatory networks inferred from these data.

Despite the maturity of available technologies to generate gene expression data, e.g., by using DNA microarrays, there is still much to learn about the capabilities of such data (Emmert-Streib & Dehmer, 2010). This is related to a variety of reasons. First, the major use of gene expression data is to identify differentially expressed genes. For this reason the majority of methods developed for these data are for this problem (Chen, Dougherty & Bittner, 1997; Ge, Dudoit & Speed, 2003; Speed, 2003; Steinhoff & Vingron, 2006; Storey & Tibshirani, 2003). Second, going beyond differentially expressed genes requires different, more sophisticated, statistical methods and the costs to generate data for, e.g., the identification of differentially expressed pathways increases substantially (Emmert-Streib & Dehmer, 2008; Reimers, 2010). Third, not only the absolute number of the available samples may be important to succeed in the application of advanced analysis methods, but also the condition and configuration used to generate the data. This last point relates to the experimental design (Hinkelmann & Kempthorne, 2008) of gene expression data used to generate these data.

In this paper, we study an aspect of the experimental design of gene expression data in the particular context of inferring gene regulatory networks from such data. Specifically, we investigate the influence of environmental conditions on the inference performance of five popular network estimation algorithms, namely, Aracne (Margolin et al., 2006), BC3NET (de Matos Simoes & Emmert-Streib, 2012), CLR (Faith et al., 2007), C3NET (Altay & Emmert-Streib, 2011; Altay & Emmert-Streib, 2010) and MRNET (Meyer, Kontos & Bontempi, 2007; Meyer, Lafitte & Bontempi, 2008). The rational behind our study is the fact that the information stored in the DNA is not sufficient to explain the phenotypic characteristics of an organism. Instead, there are genotype-environment interactions that have an important influence on this (Falconer & Mackay, 1996; Lynch & Walsh, 1998). For similar reasons studying the expression of genes without considering the environmental conditions of the cells under investigation is fragmented.

In order to study the influence of environmental conditions on the gene expression, and ultimately on the inference performance of network inference algorithms, we focus on two important, biologically relevant conditions. The first environmental condition we study corresponds to the placement of cells into a rich media. This leads to an increased proliferation of the cells due to the surplus of nutrition. The second environmental condition corresponds to a positive spike-in stimulation of cells as induced, e.g., by the administration of drugs. Here by *spike-in stimulation* we mean that the influence of a drug starts abruptly and lasts only for a short period of time. In addition to these two environmental conditions, we contrast the inference performance for data generated under these two conditions with results for data that correspond to a *normal* condition, where we do not assume an environment influence. For conducting these investigations we simulate gene expression data because this allows us controlling the corresponding conditions and simultaneously guarantees the availability of sufficiently large sample sizes to enable robust statistical findings that can be utilized to advance the experimental design of future gene expression studies aiming to infer gene regulatory networks. Specifically, for our study we generate 6600 different data sets and infer a total of 33,000 different regulatory networks.

Despite the well known fact that the environment has an influence on the expression of genes this aspect is not well studied in the literature of methods for the inference of gene regulatory networks. Instead, most studies are based on observational data only (Emmert-Streib et al., 2012). Notable exceptions in this context are studies that addressed related but different questions, e.g., investigating the appropriate level of description to simulate gene expression data, the influence of the number of time points, the number of categories and the interval length between samples (Chen, 1999; Smith, Jarvis & Hartemink, 2002; Yu et al., 2004; Husmeier, 2003). However, these studies have been conducted for time series data. Instead, in this paper we are not using longitudinal data.

This paper is organized as follows. In the next section, we describe all methods and evaluation measures we are using for our analysis. Further, we provide a detailed explanation of the data we are using and their generation. In the results section we present results for three different types of data: (I) observational gene expression data: normal environmental condition (II) interventional gene expression data: growth in rich media (III) interventional gene expression data: normal environmental condition interrupted by a brief, positive stimulation (spike-in stimulation). We study these data for five network inference methods (Aracne (Margolin et al., 2006), BC3NET (de Matos Simoes & Emmert-Streib, 2012), CLR (Faith et al., 2007), C3NET (Altay & Emmert-Streib, 2011; Altay & Emmert-Streib, 2010) and MRNET (Meyer, Kontos & Bontempi, 2007; Meyer, Lafitte & Bontempi, 2008) and two different topologies of regulatory networks. This paper finishes with a discussion and conclusions.

## Methods

In this section we describe our model, the method and the data we are using for our analysis.

### Generation of gene expression data

In order to simulate gene expression data we are using netsim (Di Camillo, Toffolo & Cobelli, 2009). Netsim is a R package that combines a fuzzy logic with differential equations to enhance the simulation of transcription regulation processes. Differential equations are used to describe the continuous dynamics of gene expression on a continuous time scale and gene-specific kinetic parameters are used to achieve realistic simulations that mimic the real dynamical behavior of gene expression. For our study we are generating gene expression data for three different conditions that correspond to two different types of data:

(I) observational gene expression data: normal environmental condition
(II) interventional gene expression data: growth in rich media
(III) interventional gene expression data: normal environmental condition interrupted by a positive spike-in stimulation

That means, we are generating gene expression data that correspond to observational (I) and interventional data (II and III). However, we are not generating data by gene knockout or silencing (Eccleston & Eggleston, 2004; Meister & Tuschl, 2004). The reason for this is that an inclusion of such perturbation experiments would limit the scope of this paper. Specifically, for human subjects it is for ethical reasons not possible to conduct *in vivo* gene-knockout experiments. Hence, if we would include such studies we would need to exclude a discussion of gene expression data, e.g., from clinical studies. On the other hand, the chosen interventional strategies for the generation of the data are equally applicable to model organisms as well as human subjects. This allows a general extrapolation of our results.

The first type of data we are generating corresponds to cells in normal environmental conditions meaning that for these simulations we do not use an external stimulation of the gene expression. The second type of data can be seen as a media rich environment which has a favorable effect on the proliferation of cells. For this condition each gene receives an external positive stimulus facilitating its expression. For your simulations this is accomplished by using a constant stimulation of a fixed positive constant *E*^*c*^. The third type of data corresponds to time dependent interventional data because we alter the environmental condition of the cells over time. This change of the environmental condition translates into a change of the dynamic of the gene expression in a time dependent manner. Specifically, we start simulating gene expression under the same conditions as in (I) but add at a certain time point, *t*_*s*_, a constant but random stimulation *E*^*s*^×*r* for each gene. Here *E*^*s*^ is a constant factor and *r* is a random variable uniformly sampled from [0,1]. This stimulation lasts a short period of time Δ*t* = 0.2. After this period, the gene expression is again governed by the same conditions as in (I). Biologically, this corresponds to a normal condition that is interrupted by a short positive stimulation, e.g., the administration of a drug.

### Interaction structure among the genes: Regulatory networks

We are conducting our analysis for two different topology types of regulatory networks that govern the interactions between genes. The first type is a Erdös–Réyni network (Erdös & Rényi, 1959; Solomonoff & Rapoport, 1951) that is generated by an algorithm. This network represents a synthetic network. The second type is a subnetwork of the transcriptional regulatory network of *S. cerevisiae* (Faith et al., 2008) and, hence, represents a real biological network. Each of these networks consists of 100 genes.

For each of these two types of regulatory networks we are generating simulated gene expression data, as described in the previous section. This allows us to study the influence that the interaction structure among the genes has on the performance of inference algorithms by keeping the dynamical system of the underlying equations unchanged.

### Simulation design of our study

**Figure 1:**
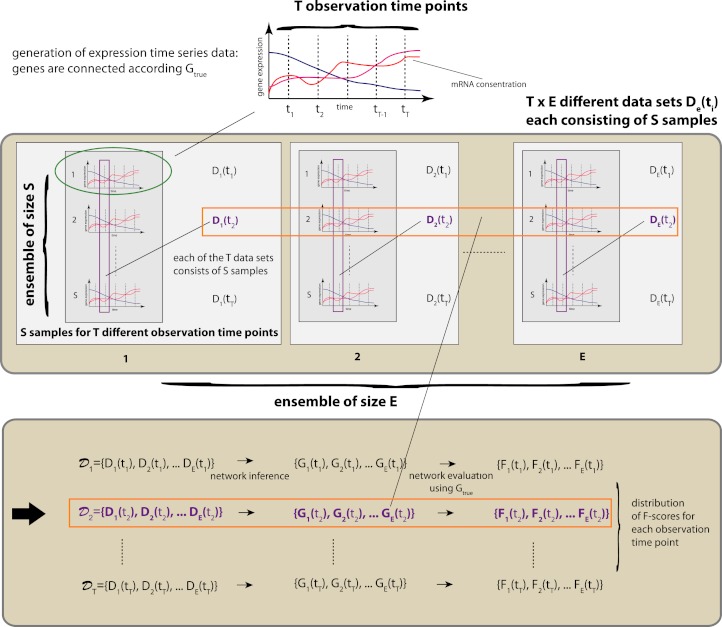
Schematic overview of our simulation design. The above procedure is repeated for each environmental condition and each *G*_*true*_ regulatory network studied.

In Fig. 1 we show a schematic overview of our simulation study. For the generation of gene expression data we are using netsim, which simulates coupled systems of differential equations. The coupling between the genes is given by a network *G*_*true*_. The connections between two genes can be positive (activator) or negative (repressor) and, hence, lead to the enhancement or repression of a transcription regulation.

We use netsim to generate time series data that are measured at *T* different time points, i.e, {*t*_1_,…,*t*_*T*_}. We are not using the time series data themselves to estimate the underlying network, given by *G*_*true*_, but, instead, we generate an ensemble of *T*×*E*×*S* different data sets. We organize these data sets according to the observation time points, i.e., 𝒟_*i*_ = {*D*_1_(*t*_*i*_),*D*_2_(*t*_*i*_),…,*D*_*E*_(*t*_*i*_)} with *i*∈{1,…,*T*}. This gives us *T* different sets of data sets, 𝒟_*i*_, each consisting of *E* different data sets *D*_*e*_(*t*_*i*_) with *e*∈{1,…,*E*} and *i*∈{1,…,*T*} with *S* samples. That means, each data set *D*_*e*_(*t*_*i*_) contains measurements that correspond to one particular time point *t*_*i*_ only. See Fig. 1 for an overview.

These sets of data sets, 𝒟_*i*_, allow us to assess the inference characteristics of statistical network inference methods on the population level, because when the value of *E* is large enough chosen it allow us to draw conclusions with respect to the behavior of the population. Specifically, we use each of the *E* data sets *D*_*e*_(*t*_*i*_) in 𝒟_*i*_ to infer *E* networks, {*G*_1_(*t*_*i*_),…,*G*_*E*_(*t*_*i*_)}. By using knowledge about the true underlying network structure among the genes, given by *G*_*true*_, we obtain *E* different F-scores that quantify the inference performance of the used network estimation algorithm, i.e., {*F*_1_(*t*_*i*_),…,*F*_*E*_(*t*_*i*_)}. Now the ensemble of F-scores allows us to estimate the mean inference performance and its variability. It is important to emphasize that information about the variability of the inference performance is necessary in order to obtain a robust evaluation. If only one or a few data sets would be used, the obtained results could be spurious. To avoid this, we use for our following numerical analysis *E* = 100, *T* = 11 and a sample size of *S* = 300. This results in a total of *T*×*E* = 1100 different data sets for each network *G*_*true*_ and each condition. Application of 5 different inference methods results in the inference of 5500 networks for each network *G*_*true*_ and each condition. In total, we infer for the two different networks we are studying (Erdös–Réyni network and subnetwork of the transcriptional regulatory network of *S. cerevisiae*) and the five different inference methods (Aracne, BC3NET, CLR, C3NET and MRNET) 33,000 different networks.

### Performance measure

In order to evaluate the performance of a network inference algorithm we are using the F-score. The F-score is defined by

(1) \begin{eqnarray*} \displaystyle F=2\frac{P\cdot R}{P+R}&&\displaystyle \end{eqnarray*}

and assumes values in [0,1], whereas zero corresponds to the worst and one to the best performance. Here *P* corresponds to the *precision* and *R* to the *recall*, i.e., 

(2) \begin{eqnarray*} \displaystyle P=\frac{T P}{T P+F P},&&\displaystyle \end{eqnarray*}

(3) \begin{eqnarray*} \displaystyle R=\frac{T P}{T P+F N}.&&\displaystyle \end{eqnarray*}

The precision and recall are functions of the number of true positives (TP), false positives (FP) and false negatives (FN). We would like to emphasize that these numbers are available from the comparison of the estimated network, *G*_*est*_, with the true network, *G*_*true*_. More precisely, for an estimated network, *G*_*est*_, the true network, *G*_*true*_, and their corresponding adjacency matrices, *A*_*est*_, and, *A*_*true*_, we obtain

(4) \begin{eqnarray*} \displaystyle T P=\sum _{i,j}I({A}_{e s t}(i,j)=1\Vert {A}_{t r u e}(i,j)=1),&&\displaystyle \end{eqnarray*}

(5) \begin{eqnarray*} \displaystyle F P=\sum _{i,j}I({A}_{e s t}(i,j)=1\Vert {A}_{t r u e}(i,j)=0),&&\displaystyle \end{eqnarray*}

(6) \begin{eqnarray*} \displaystyle F N=\sum _{i,j}I({A}_{e s t}(i,j)=0\Vert {A}_{t r u e}(i,j)=1).&&\displaystyle \end{eqnarray*}

Here *I*() corresponds to the indicator function that is 1 if its argument is true and 0 otherwise.

### Network inference methods

For our numerical analysis to infer gene regulatory networks, we use 5 different network inference methods, BC3NET, C3NET, CLR, MRNET and Aracne. In Table 1 we provide a summary of these methods. A detailed discussion of the functioning of these methods can be found in Altay & Emmert-Streib (2010), Altay & Emmert-Streib (2011), de Matos Simoes & Emmert-Streib (2012), Faith et al. (2007), Margolin et al. (2006), Meyer, Lafitte & Bontempi (2008) or in a recent review paper (Emmert-Streib et al., 2012).

All 5 methods are information theory based utilizing estimates of mutual information coefficients (Cover & Thomas , 1991). Mutual information coefficients form a non-linear extension of (linear) correlation coefficients, e.g., the Pearson correlation coefficient. Mutual information is defined by the marginal probabilities *P*(*X*) and *P*(*Y*) and the joint probability *P*(*X*,*Y*) of two random variables *X* and *Y* (Cover & Thomas , 1991):

(7) \begin{eqnarray*} \displaystyle I(X,Y)=\sum _{x_{i}\in X}\sum _{y_{j}\in Y}P(X={x}_{i},Y={y}_{j})\cdot \log \frac{P(X={x}_{i},Y={y}_{j})}{P(X={x}_{i})\cdot P(Y={y}_{j})}.&&\displaystyle \end{eqnarray*}

Here log means the logarithm to the base of 2. The mutual information, *I*(*X*,*Y*), between two random variables has the property to be always ≥ 0. *I*(*X*,*Y*) is equal to zero if the two random variables are (statistically) independent from each other, because in this case *P*(*x*,*y*) = *P*(*y*)*P*(*x*).

**Table 1:** Summary of the 5 network inference methods we use for our analysis. The first column gives the name of the method, the second provides a succinct description of the principle idea the method is based on and column three gives references describing the methods in detail.

Inference method	Principle idea	Reference
BC3NET	Bagging C3NET	(de Matos Simoes & Emmert-Streib, 2012)
C3NET	Maximal mutual information	(Altay & Emmert-Streib, 2010; Altay & Emmert-Streib, 2011)
CLR	Local estimates of mutual information	(Faith et al., 2007)
MRNET	Maximal relevance, minimum redundancy	(Meyer, Lafitte & Bontempi, 2008)
Aracne	Pairwise mutual information and DPI	(Margolin et al., 2006)

Practically, the marginal and joint probability distributions are not available and, hence, mutual information values need to be estimated by means of statistical methods from the data. In de Matos Simoes & Emmert-Streib (2011) it was found that the Miller–Madow estimator (Paninski, 2003) has overall the most favorable inference capabilities compared with 3 further esimators.

The Miller–Madow estimator utilizes the fact that the mutual information can also be written in terms of entropies (Cover & Thomas , 1991),

(8) \begin{eqnarray*} \displaystyle I(X,Y)=H(X)+H(Y)-H(X,Y).&&\displaystyle \end{eqnarray*}

Here the entropy for a random variable *X* is defined by:

(9) \begin{eqnarray*} \displaystyle H(X)=-\sum _{x_{i}\in X}P(X={x}_{i})\cdot \log (P(X={x}_{i})),&&\displaystyle \end{eqnarray*}

and the joint entropy *H*(*X*,*Y*) is given by

(10) \begin{eqnarray*} \displaystyle H(X,Y)=-\sum _{x_{i}\in X}\sum _{y_{j}\in Y}P(X={x}_{i},Y={x}_{j},)\cdot \log (P(X={x}_{i},Y={x}_{j})).&&\displaystyle \end{eqnarray*}

The simplest estimator to estimate such entropies is the empirical estimator that estimates the entropy from the observed joint frequencies for each bin (Paninski, 2003). Specifically, the empirical entropy *H*_*emp*_ can be estimated from the observed frequency distribution with *n*_*k*_ number of samples in bin *k*, the total number of samples *N* and the total number of bins *b*. For example, for the entropy in Eq. (9) the empirical estimator is given by,

(11) \begin{eqnarray*} \displaystyle {H}_{e m p}=-\sum _{k=1}^{b}\left(\frac{{n}_{k}}{N}\right)\log \left(\frac{{n}_{k}}{N}\right).&&\displaystyle \end{eqnarray*}

The Empirical estimator gives the maximum-likelihood entropy estimate for a discretized random variable. A main problem of the empirical approach is the underestimation of the true entropy, *H*, due to an undersampling of the cell frequencies when the number of bins increases. A variety of approaches have been developed to account for this bias that range from correcting the estimate by a constant factor or using a multinomial distribution to model the extend of missing information.

The Miller–Madow estimator (Paninski, 2003) accounts for the undersampling bias by adjusting the estimate by a constant factor that is proportional to the bin size and the sample size:

(12) \begin{eqnarray*} \displaystyle {H}_{m m}={H}_{e m p}+\frac{b-1}{2\cdot N}.&&\displaystyle \end{eqnarray*}

Here *b* is the number of bins and *N* is the number of samples.

A practical problem when applying the Miller–Madow estimator is that it is computationally demanding, .e.g., compared to the Pearson estimator for mutual information (Olsen, Meyer & Bontempi, 2009). The Pearson estimator for mutual information is estimated from

(13) \begin{eqnarray*} \displaystyle I(X,Y)=\frac{1}{2}\log \bigl (1-\rho (X,Y)^{2}\bigr ),&&\displaystyle \end{eqnarray*}

where ρ(*X*,*Y*) is the Pearson correlation coefficient. For normal distributed random variables *X* and *Y* this expression is exact.

From a numerical comparison of both estimators we find that the application of the Miller–Madow estimator takes about two orders of magnitude longer than the application of the Pearson estimator for mutual information. Further, from comparing different network inference methods we find that the performance for all methods is similarly effected by the estimators. For reasons of computational ease, we use for our following simulations the Pearson estimator, because our principle results are independent of the selected estimator and do not depend on the selection of the best estimator leading to the highest F-scores.

## Results

**Figure 2:**
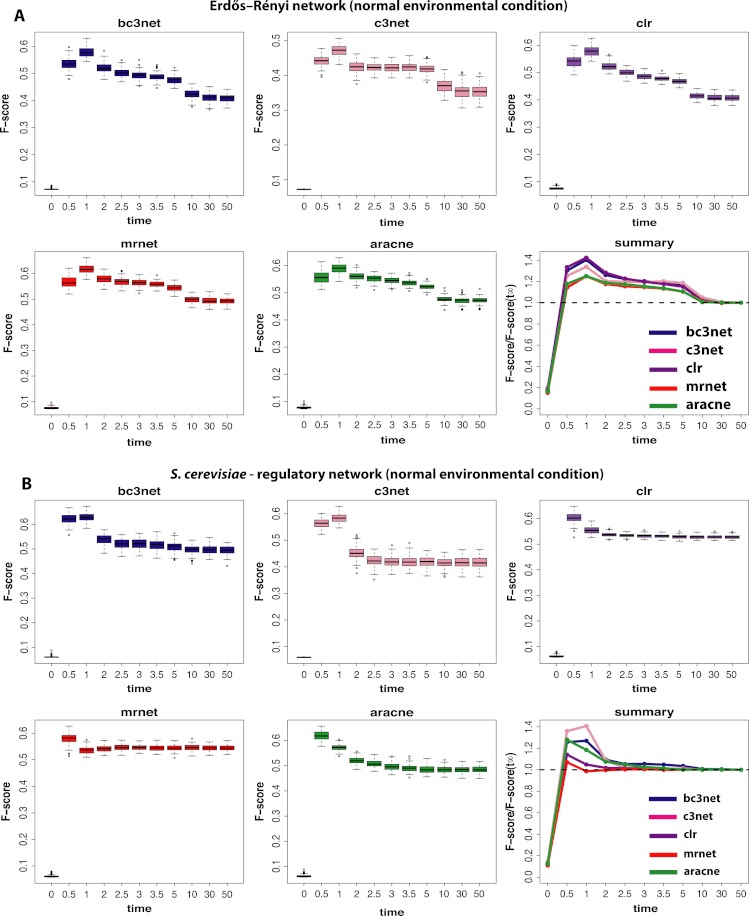
Inference performance of BC3NET, C3NET, CLR, MRNET and Aracne for a Erdös–Réyni network (A) and a subnetwork of the transcriptional regulatory network of *S. cerevisiae* (B) each consisting of 100 genes. The figures show results for *T* = 11 observational time steps, each with *E* = 100 different data sets and *S* = 300 samples. The summary figure provides information about the relative value of each F-score relative to its asymptotic value *F*(*t*_∞_).

We begin our analysis by studying data that correspond to normal environmental conditions (I). Figure 2 shows a summary of our results for BC3NET, C3NET, CLR, MRNET and Aracne. Specifically, we generate for each observational time step *t*(=(0.0,0.5,1.0,2.0,2.5,3.0,3.5,5.0,10.0,30.0,50.0)), *E* = 100 different data sets for an Erdös–Réyni network (Fig. 2A) and a subnetwork of the transcriptional regulatory network of *S. cerevisiae* (Fig. 2B). Each of these networks consists of 100 genes. That means for each time step *t*, we generate 𝒟 = {*D*_1_(*t*_*j*_),…,*D*_*E*_(*t*_*j*_)} different data sets and each of these data sets contains *S* = 300 samples (as described in section ‘Simulation design of our study’). The inference performance of each algorithm is estimated by F-scores that are presented in dependence on *t*.

From our results in Fig. 2 one can see that the F-scores of all inference methods depend crucially on the time step at which the data have been measured. For *t*_1_ = 0.0 the shown F-scores correspond to F-scores assumed by chance, because the data for *t*_1_ = 0.0 correspond to the random initial values of the underlying dynamical system used to simulate the gene expression data. As one can see, for all methods these F-scores are close to zero without being identically zero.

The long term behavior of the F-scores for all five methods, for both regulatory networks, converge to nearly constant F-scores for values of *t* larger than *t*_9_ = 10.0. This behavior indicates that the dynamical systems reach steady-state values and simulating for longer times does not lead to further changes. From our results we see that *t* = *t*_11_ = 50.0 can be safely assumed to lead to steady-state values for all five method.

**Figure 3:**
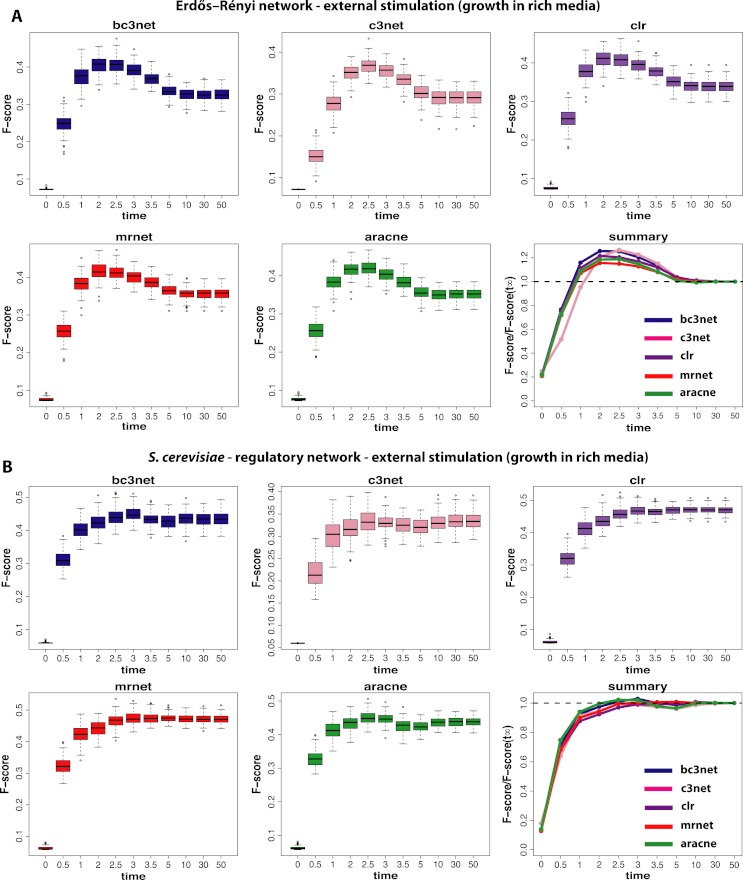
Inference performance of BC3NET, C3NET, CLR, MRNET and Aracne for a Erdös–Réyni network (A) and a subnetwork of the transcriptional regulatory network of *S. cerevisiae* (B) each consisting of 100 genes. For these data a constant external stimulation (II) has been applied.

Interestingly, the highest F-scores are observed for *t*_2_ = 0.5 and *t*_3_ = 1.0, depending on the method and the underlying regulatory network. However, in either case *t*_2_,*t*_3_≪*t*_11_, which means that the most informative time step is far from the steady-state of the dynamical system. In order to quantify the gain in the inference performance for each observational time step, we relate all median F-scores to the steady-state values, i.e., *F*(*t*_*j*_)/*F*(*t*_11_). Due to the fact that the F-scores do no longer change beyond *t*_11_, the value of *F*(*t*_11_) is equivalent to the asymptotic value of the dynamical system, i.e., $F({t}_{\infty })=\lim _{t\rightarrow \infty }F(t)$. A summary of these results is shown in Fig. 2. An interesting observation from these results is that all methods benefit from non-steady-state data by increasing their (median) F-scores by a factor of up to 1.4. However, it should be emphasized that the strength of this effect is dependent on the topology of the regulatory network, as one can see for MRNET and CLR.

**Figure 4:**
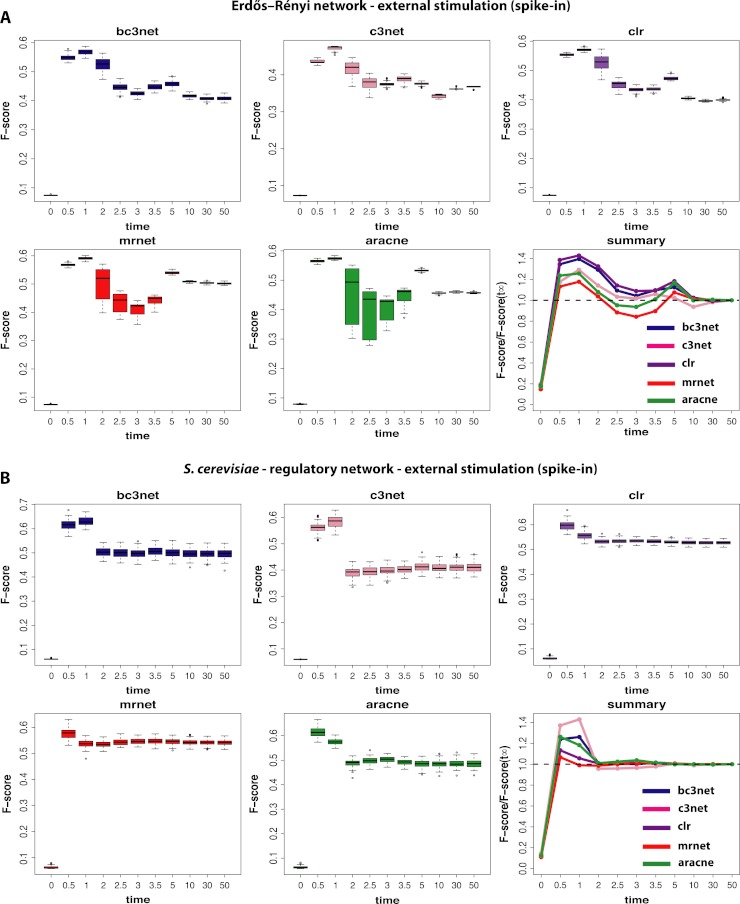
Inference performance of BC3NET, C3NET, CLR, MRNET and Aracne for a Erdös–Réyni network (A) and a subnetwork of the transcriptional regulatory network of *S. cerevisiae* (B) each consisting of 100 genes. For these data a positive spike-in stimulation (III) has been applied.

The next experimental condition we are investigating corresponds to the growth of cells in a rich media (II), as modeled by a constant and positive external stimulation, *E*^*c*^, for each gene. The results from this analysis are shown in Fig. 3. Compared to the results from the normal condition, shown in Fig. 2, there are two important differences. First, the optimal observational time step is for all methods shifted to larger values (*t*≈2.5). We repeated this analysis for different values of *E*^*c*^ and found that the larger this constant stimulation is the further one can delay the time to reach optimal F-scores. However, for too large values of *E*^*c*^ the transcription regulation is essentially driven by the external stimulation which does not lead to meaningful results.

Second, the observed results are much more sensitive with respect to the underlying topology of the regulatory network. Whereas for the Erdös–Réyni network (Fig. 3A) the overall results are similar to Fig. 2(A and B), the results for the subnetwork of the transcriptional regulatory network of *S. cerevisiae* (Fig. 3B) are qualitatively different, because now there is no gain in measuring data at time steps before the system reached its steady-state. This is consistent for all five inference methods.

Finally, we study data by simulating normal conditions interrupted by a brief period of a positive external stimulation (spike-in) (III). These results are shown in Fig. 4. The first observation is that the obtained results are again strongly dependent on the underlying network, as in Fig. 3. Additionally, we observe a method-dependent effect, because MRNET and Aracne have a considerably larger variation in the estimated F-scores for observational time steps between *t*_4_ = 2.0 and *t*_7_ = 3.5 than the other three methods. This indicates that these two methods are potentially stronger effected by the spike-in stimulation than the other methods because the simulation starts at 1.0 and lasts till 1.2. However, for all five inference methods we observe that the spike-in stimulation leads to an oscillation in the F-scores without increasing the optimal values.

For the subnetwork of the transcriptional regulatory network of *S. cerevisiae* (Fig. 4B) we find a surprising result because these results are qualitatively similar to the results for the normal condition (shown in Fig. 2B). This means that the underlying topology of the regulatory network is capable of compensating the dynamical modifications, as induced by the spike-in stimulation. Further, this behavior is method-independent because for all five inference methods, we observe qualitatively similar results.

## Discussion

In this paper we investigated the influence that environmental conditions can have on the inference performance of network inference algorithms. Specifically, we studied and compared the results for three different conditions: (I) observational gene expression data: normal environmental condition, (II) interventional gene expression data: growth in rich media, (III) interventional gene expression data: normal environmental condition interrupted by a positive spike-in stimulation. We found that different statistical inference methods lead to comparable but condition-specific results. That means, qualitatively, the five network inference methods (Aracne (Margolin et al., 2006), BC3NET (de Matos Simoes & Emmert-Streib, 2012), CLR (Faith et al., 2007), C3NET (Altay & Emmert-Streib, 2011; Altay & Emmert-Streib, 2010) and MRNET (Meyer, Kontos & Bontempi, 2007; Meyer, Lafitte & Bontempi, 2008)) we used for our study showed a similar behavior in their inference performance, for each condition. The only exception we found is for (III) interventional gene expression data (normal environmental condition interrupted by a positive spike-in stimulation) and Erdös–Réyni networks, because for this condition MRNET and Aracne assume a significantly larger variation in the estimated F-scores than the other three inference methods (see Fig. 4). However, even for this condition the observed median F-scores are for all five methods comparable.

Overall, we can draw the following conclusions from our numerical results. (1) The problem to infer gene regulatory networks from expression data is very challenging and depends on (A) the time point when data are measured, (B) the kind of the external stimulation and (C) the interconnectedness of the genes respectively their molecular interactions. Regarding the experimental design of future experiments our results suggest that it is not necessary to ensure that the gene expression data reached a stead-state value, and it could actually be detrimental for the inference of networks. Instead, usually, expression data far from the steady-state of the dynamical system contain more exploitable information that translates into increased F-scores. This finding is consistent among all five network inference methods. This makes actually the design of an experiment easier because it is practically not straight forward to control if the expression of genes reached their steady-state values. Further, for samples from human patients such a control is usually not possible for medical and ethical reasons. Hence, our findings relieve the experimenter from the need to ensure steady-state conditions in microarray experiments.

A potential explanation for this effect could be that the noise-level in the system is for the optimal time points large enough to change occasionally the expression of a gene but not too strong to shatter the concerted interaction among groups of genes. This may be comparable to the functioning of the optimization method simulated annealing (Kirkpatrick, Gellatt & Vecchi, 1983). For this method a certain among of noise (corresponding to a temperature) is necessary to overcome local minima but if the noise is too large the whole search process becomes distorted.

(2) Another important finding is that the presence of an external stimulation (as studied in this paper) did not lead to an increase in the observed F-scores. Also this finding is consistent among all five network inference methods. That means that despite the presence of a *global* perturbation on the expression of the genes this effect did not translate beneficially into an increase in the observed F-scores. This suggests that *local* perturbations or interventions need to be applied to a cellular systems in order to obtain data containing more information. For example, the knockout of genes or silencing techniques may be beneficial in this respect (Eccleston & Eggleston, 2004; Meister & Tuschl, 2004). However, the disadvantage of such interventions would be that they are for ethical reasons not applicable to human patients.

(3) Our results for (III) interventional gene expression data (normal environmental condition interrupted by a positive spike-in stimulation) and the subnetwork of the transcriptional regulatory network of *S. cerevisiae* (see Fig. 4B) hint to an intriguing design principle of gene regulatory networks. The fact that the effect of an external stimulation can be compensated by the interaction structure among genes (compare Fig. 4A with 4B) allows to raise the hypothesis that evolution might favor network structures that are less severely influenced by changes in environmental conditions. The reason for this may be an increased robustness of these systems because for different external signals the system exhibits essentially the same dynamical behavior. Previous studies investigating the robustness of gene networks focused on the elimination of interactions, (see, e.g., Jeong et al., 2000; Stelling et al., 2004; Kitano, 2007; Emmert-Streib & Dehmer, 2009; Wagner, 2005; Wagner, 2007), and not on changes of external signals, as in this study. For this reason the observed effect in our study presents a new and potentially important factor that deserves more attention in future studies.
